# Synchronized 3D Printing and Corona Charging for One-Step Prototyping of Polarized Polylactic Acid Electrets

**DOI:** 10.3390/polym15112520

**Published:** 2023-05-30

**Authors:** Zhiwei Wang, Qinghua Song, Huarui Wu, Baolong Feng, Yeyuan Li, Ling Bu

**Affiliations:** School of Information Engineering, China University of Geosciences, Beijing 100083, China; 2004220004@email.cugb.edu.cn (Z.W.); 1004215114@email.cugb.edu.cn (H.W.); 1004217123@email.cugb.edu.cn (B.F.); 1004211119@email.cugb.edu.cn (Y.L.)

**Keywords:** three-dimensional (3D) printing, electrets, surface potential, corona charging

## Abstract

Three-dimensional (3D) printing technology is advantageous in the fast prototyping of complex structures, but its utilization in functional material fabrication is still limited due to a lack of activation capability. To fabricate and activate the functional material of electrets, a synchronized 3D printing and corona charging method is presented to prototype and polarize polylactic acid electrets in one step. By upgrading the 3D printer nozzle and incorporating a needle electrode to apply high voltage, parameters such as needle tip distance and applied voltage level were compared and optimized. Under different experimental conditions, the average surface distribution in the center of the samples was −1498.87 V, −1115.73 V, and −814.51 V. Scanning electron microscopy results showed that the electric field contributes to keeping the printed fiber structure straight. The polylactic acid electrets exhibited relatively uniform surface potential distribution on a sufficiently large sample surface. In addition, the average surface potential retention rate was improved by 12.021-fold compared to ordinary corona-charged samples. The above advantages are unique to the 3D-printed and polarized polylactic acid electrets, proving that the proposed method is suitable for quickly prototyping and effectively polarizing the polylactic acid electrets simultaneously.

## 1. Introduction

Additive manufacturing (AM), also known as 3D printing, is a technique that fabricates digital models layer by layer using polymer and metal materials. It was first proposed by Charles Hull in 1986 in the stereolithography process [1]. The foundation of AM is slicing, which refers to dividing an entity into multiple fine layers of equal thickness. In detail, it requires the conversion of model files (.stl, .obj, etc.) into 3D printer action data files (.gcode) with professional software (Cura, Simplify3D, Slic3r, etc.). Ever since the technology was invented, there have been great expectations for its development in advanced manufacturing. More and more 3D printing techniques have been developed and put into practical applications, such as powder bed fusion [2], fused deposition modelling (FDM) [3], inkjet printing [4], and contour crafting [5]. The persistent innovation and progress of 3D printing technology in terms of materials, methods, algorithms, and hardware have facilitated its mature application in many fields, such as sample prototyping, part modification, item customization, etc.

The unique advantage of 3D printing lies in the fabrication of artificial materials with inner structures, such as active materials [6], composites [7], and metamaterials [8]. These artificial materials rely on the specially designed and complicated inner structures to exhibit extraordinary physical properties that are not found in natural materials. It is difficult for traditional machining methods such as lathing and milling to fabricate these inner structures. However, the characteristics of 3D printing precisely cater to this demand. For instance, electrets are important active materials with wide applications in transducers, microphones, electrometers, etc. [9]. They are dielectrics that can quasi-permanently maintain the polarization intensity after charge injection through methods such as thermal charging [10], corona charging [11], and triboelectric charging [12]. Many electrets contain cellular or porous structures, so that when external stress is applied on the materials, the holes change their sizes, resulting in voltage change across the holes. Thus, they show larger electrical constants [13].

Three-dimensional (3D) printing has been proposed to fabricate complex electrets. Taking piezoelectric electrets as an example—which have wide applications in pressure sensors, accelerometers, acoustic devices, filters, and resonators—Ikrame Najihi et al., investigated the fabrication of novel porous piezoelectric electret polymers with sealing properties and good porosity through 3D printing [14]. In their work, three kinds of piezoelectric electrets with cavity structures were fabricated, showing improved piezoelectric coefficients in the experiments. Yuri A.O. Assagra et al., proposed a one-step method to generate a piezoelectric electret polymer transducer. Taking polypropylene (PP) as the material and utilizing 3D printing techniques to accurately control the structure, they printed a two-layer film structure with regular cavity. The piezoelectric electret polymer transducer was similar to PP ferroelectrets in terms of piezoelectric coefficient and charge decay [15]. Agnieszka Mirkowska et al., explored a new layered structure containing nanoelectret materials to achieve a better piezoelectric response. They proposed a polylactic acid (PLA) mesh layer, PP electret foil, and elastomer top layer to improve the voltage response by optimizing the electrical properties and geometry of a specific layer. Among them, the PLA mesh layer was made by 3D printing, and the hexagonal holes accounted for 40% of the filling [16]. T.T.C. Palitó et al., also utilized PP as the material and superimposed two layers of extruded wires in the opposite direction through 3D printing to form the designed chess pattern structure. The adhesive copper was cut into a circular shape and glued to both sides of the film, and then it was charged with 2.5 kV DC voltage. In this design, cavities between the layers of the chess pattern structure were also realized by means of 3D printing [17]. In the study of Kierzewski et al., porous electrets were produced by 3D printing with acrylonitrile butadiene styrene. Their printing was achieved by the fused-filament fabrication technique and utilized for assembled electret structures [18].

In these studies of 3D-printing-based electrets, the model construction, material selectivity, filling mode, filling percentage, printing temperature, and other settings of 3D printing were fully utilized. These settings make the cavity structure, size, spatial density, and other geometric parameters more controllable, significantly reducing the difficulty of production. However, previous works have only explored the prototyping capability of 3D printing in fabricating electrets with specially designed structures, while they have not fully explored the polarizing capability of 3D printing in this process. In all of these studies, the charge injection was conducted after the completion of the printing, so the difficulty of charging the electrets still existed. Moreover, the separation of prototyping and charging also limits the fabrication of large-scale electret arrays. It would be more promising to charge the electrets during the printing process, i.e., prototyping and polarizing can be simultaneously realized in one step. This is conducive to charging the inner structures, which are difficult to access if charged after printing. Furthermore, considering the limited charged area adjacent to the needle tip in corona charging, including corona charging in the process of 3D printing offers the additional advantage of extending the charged area.

In this paper, we propose a synchronized 3D printing and corona charging method to achieve prototyping and polarization in one-step. The printer nozzle is modified to incorporate a high-voltage needle and apply corona charging during the printing process. The charging area restriction of corona charging is eliminated by the moving needle tip in the layer-by-layer printing process. The surface potential and charge decay performances are significantly improved due to the pre-charge injection of molten PLA. The proposed method has great potential to further enhance the accessibility of functional electrets and to foster more applications of electret-based materials, devices, and systems.

## 2. Model

**Figure 1:**
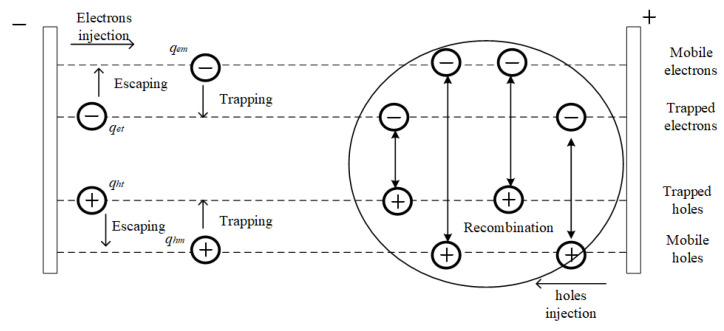
Space charge transport model of bipolar carriers.

The space charge transport model of bipolar carriers [19] was adopted to characterize the dynamic behavior of charge in polymer materials under different polarization states. In this model, the motion of the charges is mainly divided into charge injection, mobile, trapping, and escaping, as shown in Figure 1.

In this model, four different kinds of electrons are given, namely, mobile electron *q_em_*, trapped electron *q_et_*, mobile hole *q_hm_*, and trapped hole *q_ht_*. Under an electric field that is lower than 100 kV/mm, the charge injection mode is mainly Schottky injection [20]; thus, the injection current density of the mobile electron *J_em_(x,t)* and trapped electron *J_et_(x,t)* is

(1) $$J_{em}(x,t)=AT^{2}e^{-\frac{e}{K_{B}T}(\varphi_{em}+\sqrt{\frac{eE(x,t)}{4\pi \varepsilon_{0}\varepsilon_{r}})}}$$

(2) $$J_{et}(x,t)=AT^{2}e^{-\frac{e}{K_{B}T}(\varphi_{et}+\sqrt{\frac{eE(x,t)}{4\pi \varepsilon_{0}\varepsilon_{r}})}}$$

where *A* is the Richardson constant, *K_B_* is the Boltzmann constant, *T* is the temperature, *e* is the elementary charge, *φ_em_* is the mobile electron injection barrier, *φ_et_* is the trapped electron injection barrier, *E* is the intensity of the cathode electric field at position *x* and time *t*, *ε_r_* is the vacuum permittivity, and *ε*_0_ is the relative dielectric constant. Equation (1) can be utilized to describe the current density of the mobile electron *J_em_(x,t)* and trapped electron *J_et_(x,t)*.

The sum of all of the other terms in the unsteady, convection, and diffusion terms that can be included in the governing equation is the mobile electron source item *S_em_* and trapped electron source item *S_et_*, which are given by Equations (3) and (4):

(3) $$S_{em}=-R_{1}q_{ht}q_{em}-R_{3}q_{hm}q_{em}-B_{e}q_{em}(1-\frac{q_{et}}{q_{0et}})+\Gamma_{e}q_{et}$$

(4) $$S_{et}=-R_{2}q_{hm}q_{et}-R_{0}q_{ht}q_{et}-B_{e}q_{em}(1-\frac{q_{et}}{q_{0et}})-\Gamma_{e}q_{et}$$

where *R*_0_ is the recombination coefficient of the trapped electron and trapped hole, *R*_1_ is the recombination coefficient of the trapped hole and mobile electron, *R*_2_ is the recombination coefficient of the trapped electron and mobile hole, *R*_3_ is the recombination coefficient of the mobile electron and mobile hole, *B_e_* is the trapping coefficient of the mobile electron, *q_hm_* is the charge density per unit volume of the mobile hole, *q_ht_* is the charge density per unit volume of the trapped hole, *Γ_e_* is the trapped electron’s escaping coefficient, and *q*_0*et*_ is the trapped electron’s density.

Finally, the current equation is utilized to describe the distribution of mobile electrons and trapped electrons at time *t* in position *x* with the current density and source item in Equations (1)–(4), as shown in Equations (5) and (6):

(5) $$\frac{\partial q_{em}(x,t)}{\partial t}=S_{em}(x,t)-\frac{\partial J_{em}(x,t)}{\partial t}$$

(6) $$\frac{\partial q_{et}(x,t)}{\partial t}=S_{et}(x,t)-\frac{\partial J_{et}(x,t)}{\partial t}$$

Specifically, temperature is a variable in the process of 3D printing in the proposed method. The temperature of the PLA material in the molten state is 483 K. During the extrusion process, the PLA material is in a vitrified state, and its temperature is between 332 K and 442 K. Compared with ambient conditions, taking 298 K as an example, the charge injection temperature in the proposed method is higher. It can be seen from Equation (1) that the injection current density is positively correlated with the temperature. Typically, in the corona charging process, the electric field intensity at the needle tip reduces with increasing distance; thus, the charge inside the sample is reduced. However, the high temperature increases the charge inside the sample at the position of weak electric field intensity. Moreover, it was mentioned in the work of Galikhanov [21] that preheating has an influence on the charge injection of PLA material. After preheating, the dielectric coefficient will decrease and the relaxation time will increase.

## 3. Methods

The existing method is to first 3D print the electrets, followed by corona charging, encompassing two separate steps. However, the proposed method combines printing and corona charging in one step. When the prototype fabrication is completed with the 3D printer, the charging injection is also completed.

In the experiment, the 3D printer was a hori Z500 (HORI, Beijing, China), which is based on the FDM technique. FDM has been widely used in AM, having been developed by Stratasys Inc. in the USA [22]. In the operation process, materials are transported by a stepping motor into the heating block of the sprinkler head, and then they are electrically heated and melted into a liquid state when the temperature reaches 483.15 K. Then, the material is extruded. This process mainly relies on the thermoplasticity of the polymer. According to the calculated printing path, the extruded filamentous material can be fused with the previous layer of material, and the material can be solidified under ambient conditions. Eventually, the printing task is completed.

In the selection of materials, we adopted PLA as the printing material. Although its polarization effect may be limited compared with fluoropolymers [21], 3D-printed PLA has stronger mechanical properties, including elastoplastic and orthotropic behavior, as well as compression and tension asymmetry [23]. The physical properties of PLA are illustrated in Table 1, and the process variables are illustrated in Table 2.

Instead of a fully filled structure, the interior is filled by a diamond shape with a side length of 5 mm. By utilizing the software Magics, which analyzes the digital model layer by layer, the model slicing and print parameter input can be finished.

**Figure 2:**
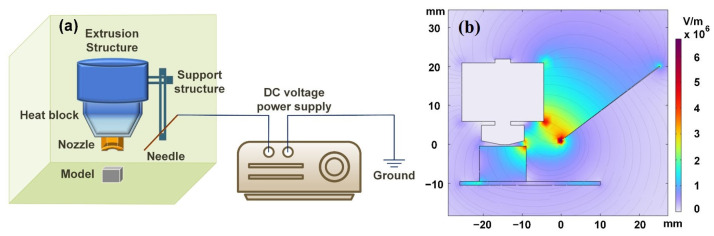
(**a**) System configuration for synchronized 3D printing and corona charging. (**b**) Electric field intensity in the finite element model.

As is shown in Figure 2a, the one-step printing device consisted of a 3D printer and a high-voltage DC power supply, each of which was grounded by its own ground wire.

We applied corona charging to polarize the in-print material. We fixed the needle on the supporting rod of the base through the pinhole and wrapped it with insulation tape, so as to fix it to the nozzle. The distance between the needle tip and nozzle could be adjusted by the fixed structure. The charge was injected into the in-print material by placing the needle tip at a fixed distance from the nozzle, and the corona charging was conducted on the needle tip through a high-voltage DC power supply of the given voltage. Different voltages were controlled by the high-voltage DC power supply. The charging time was set to be the same as the model printing time. A finite element model is shown in Figure 2b that illustrates the electric field intensity in the printing process. The electric field intensity between the heat block and the tip was greater than the minimum electric field intensity of 3 MV/m, which would cause air breakdown. We applied a −8 kV voltage for the pre-test. When the needle tip distance was less than 16 mm, air breakdown occurred between the needle tip and the nozzle, resulting in undesired discharge. It is consistent with the finite element model. Therefore, we set 16 mm as the lower limit of the needle tip distance and −8 kV as the upper limit of the applied voltage.

After the electret printing was completed, we utilized a surface potentiometer (MODEL 344-K, TREK INC, Lockport, NY, USA) to measure the surface potential of the electrets. The printed sample was placed in the center position of the grounded test stand. The distance between the probe of the device and the sample surface was fixed at 2 mm. The position of surface potential measurement was adjusted through the X-Y stage.

## 4. Results and Discussion

### 4.1. Printer Nozzle Refitting

**Figure 3:**
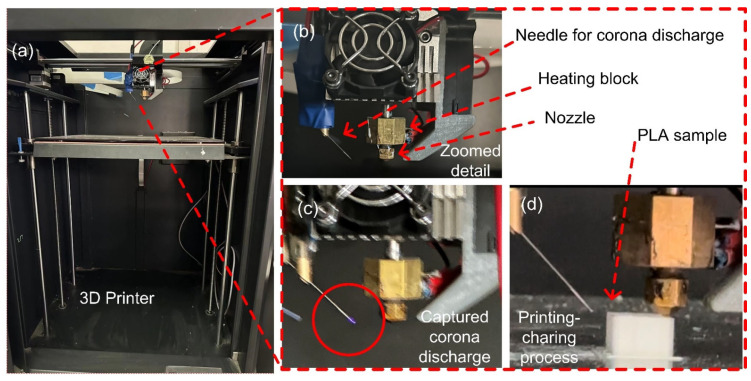
Photos of the 3D printer and printing/charging process: (**a**) Photo of the 3D printer. (**b**) Enlarged photo showing the high-voltage needle adjacent to the printer nozzle. (**c**) Enlarged photo of the captured corona discharge. (**d**) Enlarged photo of the printing/charging process.

As shown in Figure 3a, we injected the charge into the sample by corona charging while printing it.

In the experiment, we applied −6 kV, −7 kV, and −8 kV voltages under the needle tip distances of 16 mm, 18 mm, and 20 mm, respectively. Additionally, we applied a −6 kV voltage under the needle distance of 17 mm for large-area surface potential measurement. Due to the small curvature of the tip, when the high-voltage DC source was switched on, corona charging was generated at the needle tip and ions were released. The charge moved towards the surface of the model under the electric field and gradually migrated to the sample. Eventually, they were captured and accumulated in the PLA sample.

### 4.2. SEM Photos of Printed Samples

**Figure 4:**
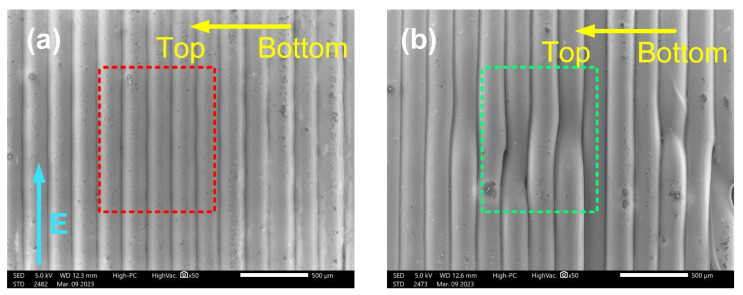
SEM photos: (**a**) 3D printing with corona charging; (**b**) 3D printing without corona charging.

The layered structure of the printed samples can be observed in the scanning electron microscopy (SEM) photos in Figure 4. As a comparison, Figure 4a shows an SEM photo of the PLA sample surface with synchronized 3D printing and corona charging, while Figure 4b shows that of the PLA sample directly printed without corona charging. In both subplots, the yellow arrow marks the printing direction from the bottom to the top, and the blue arrow marks the direction of the electric field exerted by the corona charging. The red dashed box in Figure 4a and the green dashed box in Figure 4b both show the printed layers of the PLA samples. Twisted and sunken layers appear in the green dashed box where no electric field was applied, while the layers are straight in the red dashed box where the electric field was applied. Comparing these two parts, the corona charging electric field contributes to keeping the layers straight in the printing process and reduces the appearance of non-ideal twisted and sunken layers.

### 4.3. Surface Potential Measurement

**Figure 5:**
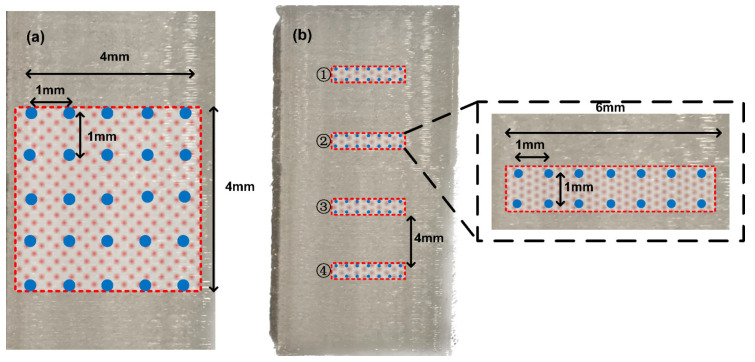
(**a**) The 5 × 5 point surface potential measurement region in the center of the sample. (**b**) Four surface potential measurement regions with 4 mm intervals, each containing 7 × 2 points.

Figure 5 shows the printed sample surface and marks the area where we measured the surface potential. Figure 5a shows the measurement region in the central part of the PLA sample, consisting of 5 × 5 points with 1 mm gaps both horizontally and vertically. The total 4 mm × 4 mm measurement region is for characterization of the spatial distribution of the surface potential. To demonstrate that the proposed method is also suitable for wide regional charging, in Figure 5b, four rectangular measurement regions with 4 mm gaps are marked. The entire PLA sample surface is divided into four parts, and the center of each part is selected as one of the four measuring regions. The four regions are marked as ①~④, each consisting of 2 × 7 testing points. Each testing point is also 1 mm adjacent to the surrounding testing points.

**Figure 6:**
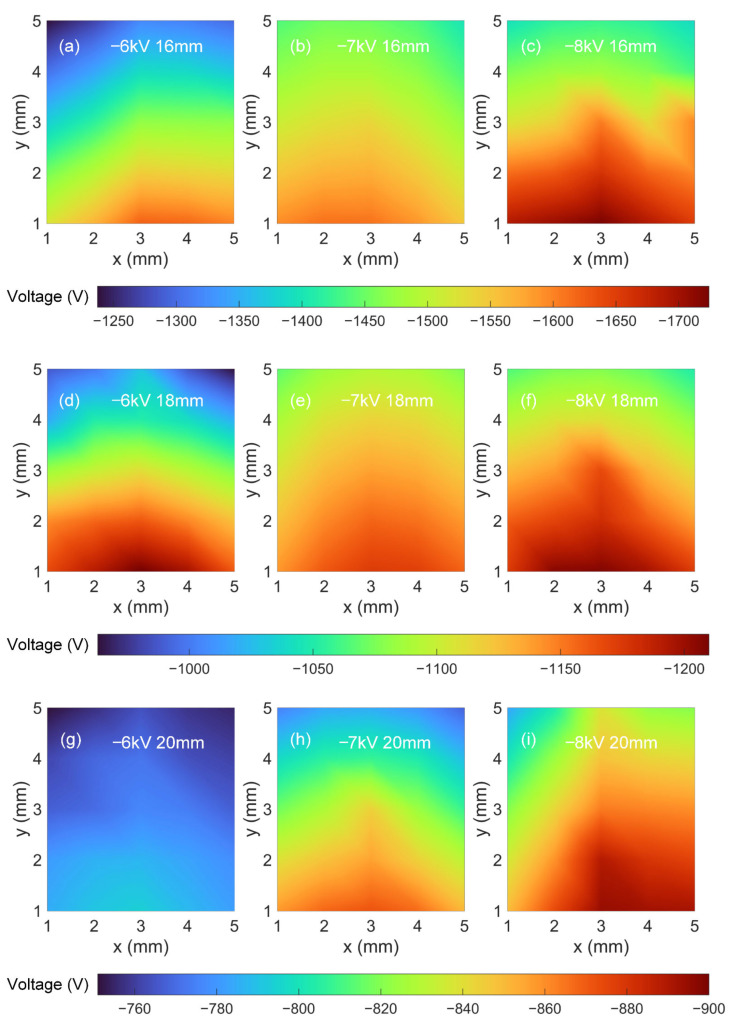
Measured surface potential contours under different charging voltages and needle tip distances. The measurement regions are consistent with Figure 5a.

**Figure 7:**
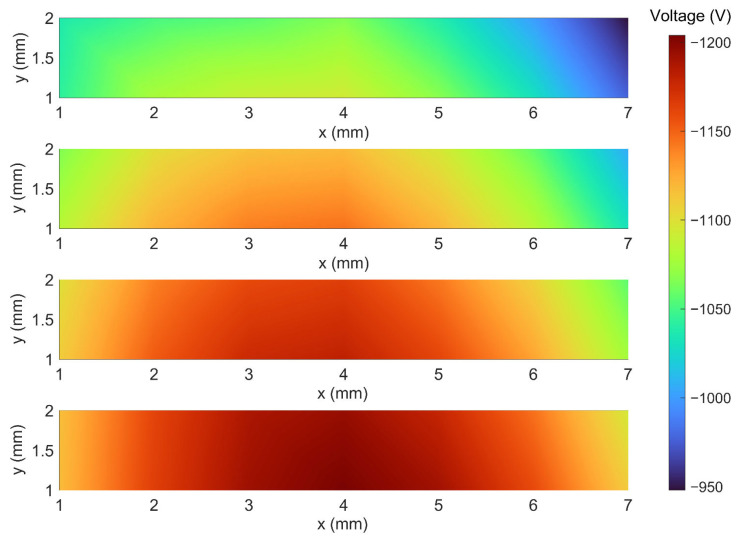
Measured surface potential under −6 kV and 17 mm. The four measurement regions are in accordance with Figure 5b.

Figure 6 shows the surface potential under different corona charging voltages (−6 kV, −7 kV, and −8 kV) and needle tip distances (16 mm, 18 mm, and 20 mm). The measured surface potential contour under −6 kV and 17 mm is shown in Figure 7, where No. ①~④ corresponds to the measurement regions ①~④ in Figure 5b, respectively. In the ordinary corona charging method, the distribution of surface potential decreases from the center to the outside, typically resulting in a bell-shaped distribution of surface potential. However, in the proposed printing and charging method, the distribution of surface potential can be extended to a sufficiently wide region beyond the limit of the needle tip position. Following the nozzle’s movement trajectory, the needle tip moves with the printer nozzle, covering a wider range across the sample surface and spending a longer time charging. This also contributes to mitigate the traditional bell-shaped distribution of surface potential in corona charging, resulting in a more uniform surface potential distribution. Consequently, the surface potential ranges in regions ①~④ are [−948 V, −1090 V], [−1006 V, −1145 V], [−1056 V, −1181 V], and [−1095 V, −1204 V], respectively. It can be seen that a high level of surface potential is achieved in all four regions, proving that the proposed method can conduct large-surface corona charging.

**Figure 8:**
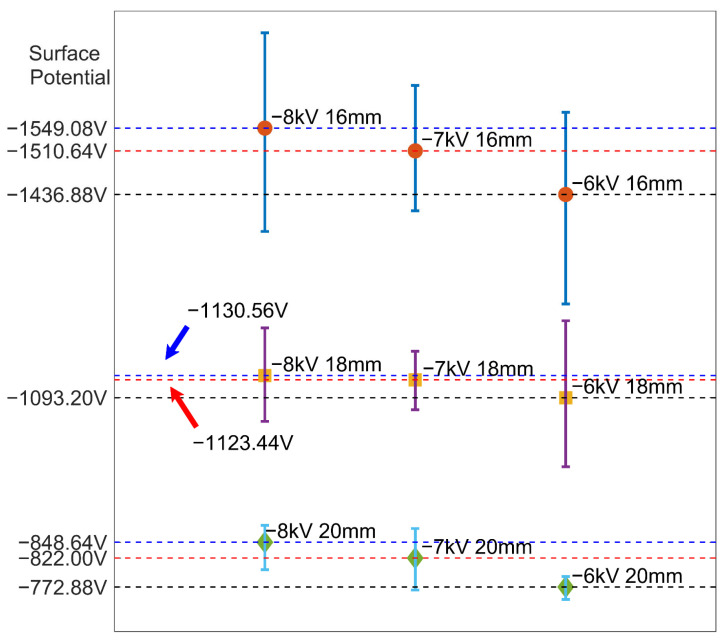
Average surface potential under different conditions.

Figure 8 summarizes the differences in surface potential distribution under different charging conditions. We calculated both the mean value and the standard deviation of the surface potential in each case. Relatively uniform charge distribution could be achieved on a sufficiently large sample surface, with the standard deviation of surface potential not exceeding 36.98%. Generally, the surface potential decreases as the charging voltage decreases. The mean values of surface potential under different conditions in Figure 8 support the conclusion in Figure 6 that the influence of the charging voltage on the surface potential level is much smaller than that of the needle tip distance. Similarly, the uniformity comparison also shows that the charging voltage has almost no influence on the uniformity of the surface potential distribution. When the needle tip distance is 20 mm, regardless of the charging voltage, the surface potential distribution shows good uniformity. However, the uniformity of the surface potential distribution deteriorates as the needle tip distance decreases. The correlation between the uniformity of the surface potential distribution and the needle tip distance can be explained from the perspective of electric field intensity. With a large needle tip distance, the electric field at the sample surface is smaller, but the distribution of the electric field is more uniform. Although the needle tip moves continuously in the printing process, the variation in the electric field intensity at the sample surface is small. Conversely, when the needle tip distance decreases, the inhomogeneous distribution of the electric field on the sample surface leads to a high electric field intensity at the position that is horizontally close to the needle tip in the path of the moving needle tip. Therefore, the variation in the electric field intensity at the sample surface is large and results in large non-uniformity in the surface potential distribution.

### 4.4. Surface Potential Decay Analysis

**Figure 9:**
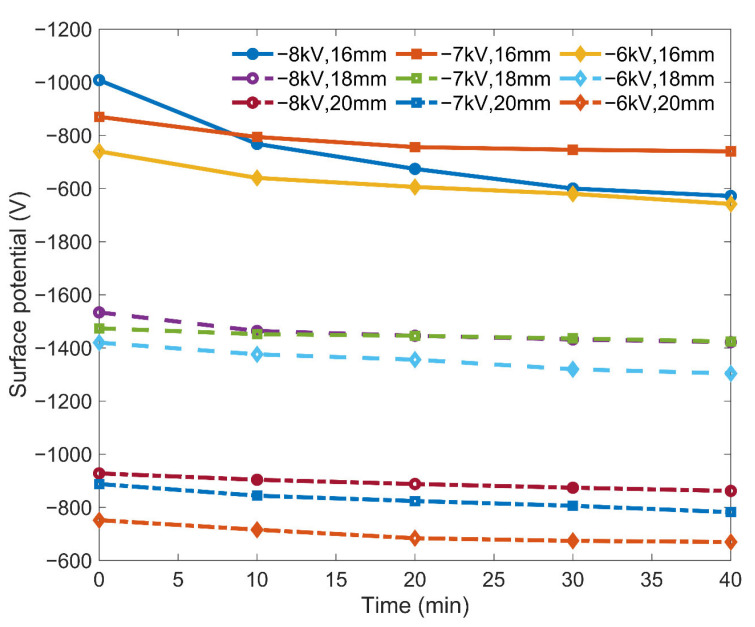
Surface potential decay.

Finally, the surface potential decay at the center point in Figure 5a was recorded every 10 min for 40 min, and the corresponding curves are shown in Figure 9. Under the voltages of −8 kV, −7 kV, and −6 kV, the average surface potential decay ratio within 40 min was 7.40%, 4.24%, and 5.75%, respectively. Under the condition that the needle tip distance was 16 mm, 18 mm, and 20 mm, the average surface potential decay ratio within 40 min was 8.18%, 4.08%, and 5.13%, respectively. This shows that the surface potential decay rate of the sample was low within 40 min. The surface potential experienced a large decay in the first 10 min and then gradually approached a stable value in the following 30 min. This feature remained the same for different corona charging voltages at −6 kV, −7 kV, and −8 kV, and for the different needle tip distances of 16 mm, 18 mm, and 20 mm. Such characteristics show that the charges decay faster in the early stage than in the later stage, because of the detrapping of charges at different energy levels with variant tangential electric field intensity. In the early stage, the charges of the shallow trap level detrap, and the tangential electric field is strong, causing a fast decay rate of the surface potential. In the later stage, the charges of the deep trap level detrap, and the tangential electric field is weak, causing a slow surface potential decay rate.

According to the surface potential decay results, the surface potential of our samples has good retention. To make a further comparison, double exponential fitting was performed on all of the surface potential decay curves in Figure 9, and on the surface potential decay curve of the PLA sample that was charged after printing for comparison. The fitting results are given in Table 3.

We obtained the time constant *τ*_1_ to describe the surface potential decay rate in the early stage and the time constant *τ*_2_ to describe it in the later stage. As demonstrated in Table 3, for *τ*_1_, the synchronously charged and printed sample and the sample charged after printing had similar values, showing that the proposed method has little impact on the surface potential decay in the initial stage. However, in the later stage, the time constant *τ*_2_ of the synchronously charged and printed sample was much greater than that of the charged sample after printing, ranging from 5.40 to 32.94 times higher. On average, the *τ*_2_ of the synchronously charged and printed sample was 101.83901, which is 13.02121 times higher than that of the sample charged after printing.

These results prove that the proposed method is advantageous not only in one-step prototyping of polarized PLA, but also in obtaining PLA electrets with highly stable trapped charges. In the work of [24], the obtained initial surface potential was in the range of 850 V to 900 V. By fitting the surface potential decay, the time constant was in the range of 30.77 to 40.42. It can be seen that the proposed method has at least a 32.79% improvement in surface potential decay. Comparing the initial surface potential and the surface potential decay of the proposed method, the electrical performance of PLA is improved by the proposed method.

## 5. Conclusions

This paper proposed a solution for synchronously prototyping and polarizing electrets based on an FDM 3D printer, with PLA as the printed material. The method was theoretically explained by a space charge transport model of bipolar carriers. The core of this method is to incorporate a high-voltage charging device with the 3D printer, which conducts charge injection during the prototype printing. The proposed method has high extendibility to different types of 3D printers. What needs to be considered is the air breakdown between the needle tip and the nozzle, which can be solved by controlling the needle tip distance or adjusting the applied voltage. By incorporating a needle electrode to apply a high voltage, the polylactic acid electrets can be printed and corona-charged in one step. The sample characterizations show the following:

- Relatively uniform charge distribution can be achieved on a sufficiently large sample surface, with the standard deviation of surface potential not exceeding 36.98%;
- The surface potential retention rate is improved by 5.40~32.94-fold compared to ordinary corona-charged samples;
- The proposed method has at least a 32.79% improvement in surface potential decay;

The proposed method shows the possibility of charging large-volume samples with corona charging, and it broadens the application of 3D printing in electret manufacturing. In future research, the influence of the external electric field on the printing process of FDM-based 3D printing requires further verification. Meanwhile, the feasibility of multi-angle charging of samples with a movable high-voltage needle connected to the nozzle is also worth exploring.

## Figures and Tables

**Table 1:** Physical properties of PLA.

Physical Properties of PLA	Value
Density	1.25 g/cm^3^
Melting point	449.15 K
Intrinsic viscosity	1.20 dL/g
Glass transition temperature	333.15 K
Tensile strength	40.00 MPa
Elastic modulus	3000.00 Mpa
Flexural modulus	100.00 Mpa
Dielectric dissipation factor	0.01
Relative permittivity	3.10
Volume resistivity	4.30 × 10^17^ Ω·cm

**Table 2:** Process variables in the experiment.

Process Variables	Value
Length of cuboid sample	12 mm
Width of cuboid sample	12 mm
Height of cuboid sample	30 mm
Filling percentage	10%
Printing speed	75 mm/s
Printing temperature	483.15 K

**Table 3:** Time constant of surface potential decay under different corona charging conditions.

Charging and Printing	Time Constant *τ*_1_	Ratio	Time Constant *τ*_2_	Ratio
−8 kV, 16 mm when printing	0.62344	97.8%	58.65102	749.9%
−7 kV, 16 mm when printing	0.90991	142.6%	265.46323	3394.2%
−6 kV, 16 mm when printing	0.03631	5.7%	55.18763	705.6%
−8 kV, 18 mm when printing	0.30525	47.9%	124.25447	1588.7%
−7 kV, 18 mm when printing	0.03888	6.1%	150.76134	1927.6%
−6 kV, 18 mm when printing	0.03671	5.7%	53.67686	686.3%
−8 kV, 20 mm when printing	0.64184	100.6%	89.84725	1148.8%
−7 kV, 20 mm when printing	0.03602	5.6%	50.07511	640.2%
−6 kV, 20 mm when printing	0.36805	57.7%	68.63417	877.6%
−7.7 kV, 20 mm after printing	0.63775	100.0%	7.82101	100.0%

## Data Availability

Data can be obtained by emailing lingbu@cugb.edu.cn for acceptable reasons.
